# Use of blood components in critically ill patients in the medical intensive care unit of a tertiary care hospital

**DOI:** 10.4103/0973-6247.53879

**Published:** 2009-07

**Authors:** R N Makroo, R. K. Mani, Raina Vimarsh, Sudha Kansal, Kumar Pushkar, Sandeep Tyagi

**Affiliations:** *Departments of Transfusion Medicine, Indraprastha Apollo Hospitals, Sarita Vihar, New Delhi -110 076, India*; 1 *Department of Respiratory Medicine, Indraprastha Apollo Hospitals, Sarita Vihar, New Delhi -110 076, India*

**Keywords:** Fresh frozen plasma, medical intensive care unit, packed red cells

## Abstract

**Background:**

The art of fluid administration and hemodynamic support is one of the most challenging aspects of treating critically ill patients. Transfusions of blood products continue to be an important technique for resuscitating patients in the intensive care settings. Concerns about the rate of inappropriate transfusion exist, particularly given the recognized risks of transfusions and the decreasing availability of donor blood. We investigated the current transfusion practice in the critically ill patients at our hospital.

**Materials and Methods:**

A total of 1817 consecutive critically ill patients admitted between January 2006 and December 2006 were included in this retrospective study. The blood request forms of the patients were analyzed, and their pretransfusion investigations, indications for transfusions, etc. were studied.

**Results:**

Nine hundred and eleven (50.1%) critically ill patients, comprising 71.6% males and 28.4% females, received blood/blood components. About 43.8% patients were administered packed red cells (PRC), 18.27% fresh frozen plasma (FFP) and 8.4% transfused platelets. Among those receiving PRC, 31.1% had a pretransfusion Hb below 7.5g%, 34.4% had Hb between 7.5 and 9g%, while 21.4% had Hb above 9g%. Among those receiving FFP, 14.5% had an international normalized ratio INR < 1.5, and 19% had a pretransfusion platelet count above 50,000/cumm. During the study, there were 7% of the patients who received red cells and FFP, 2% of the patients received red cells and platelets, 1% of the patients received platelets and FFP, and 5% of the patients had received all the three components, i.e., red cells, FFP and Platelets. The baseline investigations and/or clinical indications were not mentioned in 13.1% of patients receiving PRC, 57% receiving FFP and 49.7% receiving platelets.

**Conclusion:**

About 21.4% of PRC, 14.5% of FFP, and 19% of platelets were inappropriately indicated. Clinicians in our centre were conservative in keeping with recent transfusion guidelines. A significant number of blood request forms were still incomplete with baseline investigations not mentioned in the request forms.

## Introduction

Transfusion of blood and blood components plays an important role as supportive therapy in critically ill patients. It has been estimated that one-third of all patients admitted to intensive care units in the developed world receive blood transfusions.[1] In the past, hemoglobin concentration was more often maintained above 10 g/dl in the critically ill in the belief that high oxygen carrying capacity and oxygen delivery were important to prevent tissue hypoxia and organ failure.[2,3] This practice has been established by clinical trials that showed that maintaining supranormal oxygen delivery in patients with established critical illness does not change outcome[4] and that clinically resuscitated critically ill patients do not often exhibit pathological oxygen supply dependency.[5‐7] This retrospective study was carried out to evaluate red cells, platelets and plasma transfusion practices in the medical intensive care units in a tertiary care hospital in New Delhi.

The aim of the study is to find out the current transfusion indications, pretransfusion hemoglobin concentrations, pretransfusion INR and pretransfusion platelet counts in critically ill patients who required > 24 h of stay in the medical intensive care units (ICUs)

## Materials and Methods

The study took place in the ICUs (Medical ICU, Cardiac Care Unit and Gastro Intensive Care Unit) of the Indraprastha Apollo Hospital, New Delhi, which is a 694-bedded tertiary-referral centre and annually admits about 2000 patients in the Medical ICUs. The study was carried out for 12 consecutive months from January 2006 to December 2006. Bed occupancy in Medical ICUs was 91% and >90% of patients received mechanical ventilation. The study was conducted mainly for the medical cases. The patient in the study group remained in the ICUs for more than 24 h.

For red cell transfusion, the patients were categorized into four groups based on the pretransfusion hemoglobin concentration, i.e., <7.5g%, 7.5–9g% and >9g% and forms where hemoglobin (Hb) was not mentioned. The frequency, indications and timing of red cell transfusion events in relation to ICU admission were described. There were mainly two types of indications: i) Hemorrhage: the patient required transfusion because of recent/ ongoing clinically evident blood loss. ii) “Reduced physiological reserve”: the patient was not bleeding or losing blood through other clinically apparent mechanism.

For plasma transfusion, the patients were categorized on the basis of INR i.e., < 1.5 and > 1.5 and INR not mentioned. For platelet transfusion, the patients were categorized into four categories based on pretransfusion platelet counts, i.e., Category I: <20,000/cmm with no associated bleeding, Category II: 20,000–50,000/cmm with associated bleeding, Category III: >50,000/cmm, and Category IV: – platelet count not mentioned.

The blood request forms of the patients were analyzed, and the various parameters and the blood or blood components issued were studied.

## Results

A total of 1817 patients were admitted in Medical ICUs from January 2006 to December 2006. Of these, 1269 (69.8%) were males and 548 (30.2%) were females. The total number of patients who received blood/blood components transfusion was 911 (50.1%), out of which 652 (71.6%) were males and 259 (28.4%) were females [Table 1]

**Table 1:** Number of patients receiving blood / blood component transfusion among those admitted in MICU

	No. of admissions in the MICU during Jan 2006-Dec 2006	No of patients receiving blood/blood component transfusion
Males	1269	652
Females	548	259
Total	1817	911

**Figure 1:**
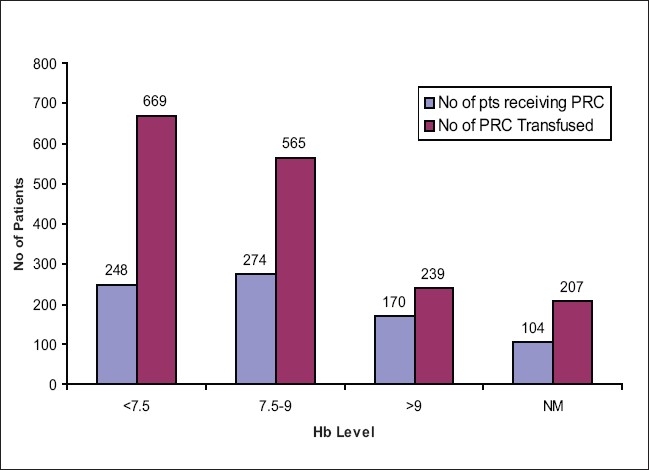
PRC transfusion in different Hb parameters in MICU patients

**Figure 2:**
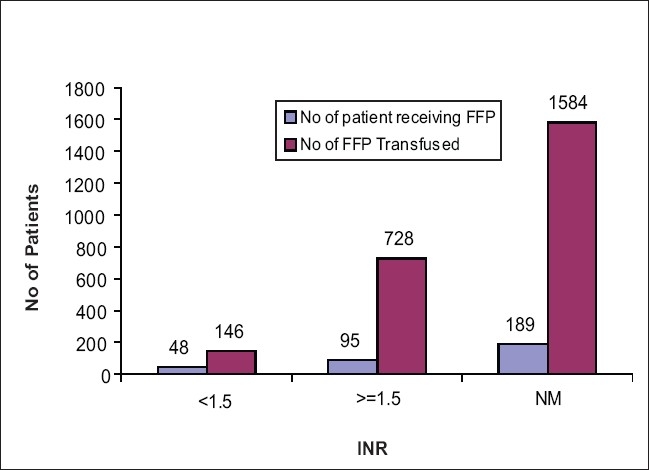
Number of patients receiving FFP in MICU

Packed red cells (PRC) were transfused to 796 (43.8%) patients, with 248 (31.1%) of them having a pretransfusion Hb levels below 7.5 g/dl, 274 (34.4%) of them had pretransfusion Hb levels between 7.5 and 9 g/dl, 170 (21.4%) had above 9 g/dl, and in 104 (13.1%) patients, Hb was not mentioned [Figure 1]. Fresh frozen plasma (FFP) transfusion was given to 332 (18.27%) patients. Forty-eight patients (14.46%) had pretransfusion INR levels below 1.5. Ninety-five (28.61%) had pretransfusion INR levels more than 1.5. The INR levels of the remaining 189 patients (57%) were not available [Figure 2]

**Figure 3:**
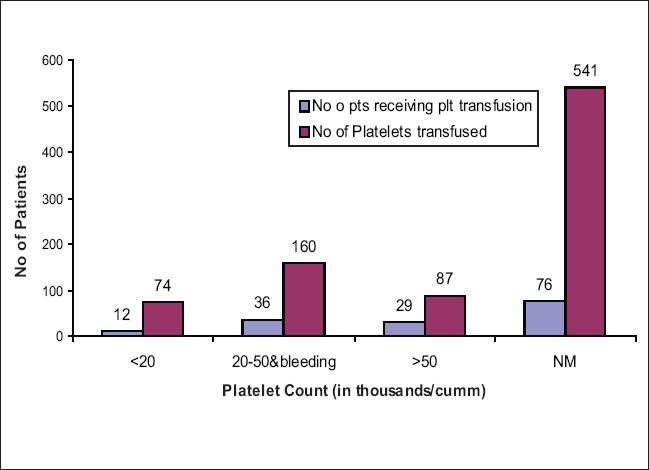
Number of patients receiving platelets transfusion in MICU

A total of 153 patients (8.4%) received platelet transfusion. 7.8% of them had pretransfusion platelet counts below 20,000/cmm. 23.5% of them had pretransfusion platelet counts between 20,000 and 50,000/cmm and bleeding. 19% of the patients had platelet counts above 50,000/cmm and in 49.7% patients, the platelet values were not mentioned [Figure 3] During the study, there were 128 patients (7%) who received red cells and FFP. Thirty-nine patients (2%) received red cells and platelets. Nineteen patients (1%) received platelets and FFP. There were 92 patients (5%) who had received all the three components, i.e., red cells, FFP and platelets.

## Discussion

A substantial proportion of blood transfusion occurs in the ICUs in the critically ill patients. Corwin, in his study, found that by the third day of a patients stay in the ICU, more than 90% of patients were anemic.[8] As many as 85% of patients with an ICU length of stay more than 7 days received at least 1 unit of blood, and on an average, these patients received 9.5 units during their ICU stay.[9] In the 1999 Canadian Clinical Trials Group study, it was suggested that there is no clinical benefit in maintaining a hemoglobin level greater than 100 g/l in critically ill patients. In the absence of cardiac dysfunction or critical coronary artery disease, a hemoglobin concentration of 8 g/dl adequately meets the oxygen needs of most patients.[10] Most tissues that are adequately perfused will not become ischemic even with a hemoglobin concentration as low as 7 g/dl.[11]

The study conducted by us in the ICU of the hospital showed the following facts. Clinicians in our centre were conservative in keeping with recent transfusion guidelines. The level of 10 g/dl has been used as a transfusion trigger for many years for surgical and leukemic patients, which has now been lowered to 7.5 g/dl.[12] With the lowering of transfusion trigger to 7.5g%, a lot of unnecessary transfusions have decreased. However, 21.4% of the patients who received PRC had a pretransfusion Hb level above 9 g/dl and this may be because the transfusion trigger is not the sole criteria for blood transfusion as the overall condition of the patient has to assessed by the physician and then it has to be decided whether or not blood transfusion is required. To improve the blood transfusion services (BTS) in the hospital, a regular communication between the treating physician and the Department of Transfusion Medicine has been given utmost importance. This not only keeps us informed about the patient’s condition but also makes us prepared in case of an emergency blood transfusion. To a large extent, this has prevented unnecessary or over-enthusiastic blood transfusions in our centre.

There were a significant number of patients (14.5%) whose pretransfusion INR was below 1.5 but received FFP transfusion. This was mainly due to the total plasma exchanges (TPE) done on few of them who were diagnosed with thrombotic thrombocytopenic purpura, myasthenia gravis, Goodpasture’s Syndrome, etc. The usage of FFP show that a significant number of patients received FFP for indications that fit the recommendations in the British Committee for Standards In Haematology (BCSH) guidelines.[13] Although 14.5% of patients appeared to have been given FFP for reasons not clearly specified within the guidelines, this does not necessarily imply that the use was inappropriate. Clinical details obtained during the audit were sometimes incomplete.

In other cases, the result of coagulation tests not available before the administration of FFP was considered necessary by the clinicians managing the patient.

However, the results of the audits do suggest that FFP is still sometimes over-prescribed and strict clinical criteria for the demand of FFP from hospital blood banks needs to be enforced. To establish an appropriate use of FFP in our center, we have a policy in our hospital that all FFP requests needs to be sufficed with the indications for FFP as well as the patients’ Partial Thromboplastin Time (PT)/ Activated Partial Thromboplastin Time (APTT) and International Normalized Ratio (INR) values. In case, the form does not carry the same information, the concerned doctors/nurses are contacted, and the necessary information is obtained. This has by and large reduced the wastage and the over-prescription of FFP.

About 19% of platelet transfusions were given at values in the order of 50-100 × 10^9^/L. The pretransfusion platelet count varied according to the indications for transfusion, e.g., cases of sepsis or patients undergoing minor surgical procedures. A significant finding in our studies was that a significant percentage of blood request forms were incomplete. Since a lot of these requests are marked as emergency, the blood bank personnel issue these blood products even in the absence of baseline investigations. However, after the blood or blood component is issued, the clinical audit is done to assess the cause of the incomplete request. Furthermore, the concerned doctor is also informed and educated about the need for filling the request form completely in the hospital transfusion committee (HTC) meeting. The regular interaction between the clinician and our department with regular follow up has made a significant decrease in the number of incomplete request forms as most of them are duly filled at present.

About 22.3% PRC and 23.1% of FFP transfusion orders were inappropriate in a study done by Kakkar *et al*. in 2003.[14]The newer trend in critical care medicine is the recent surge in randomized, controlled trials designed to test the validity of commonly used diagnostic and therapeutic practices. Organizations such as the Canadian Critical Care Trials Group, the National Heart, Lung and Blood Institute through its Acute Respiratory Distress Syndrome Clinical Trials Network and several European consortiums are working systematically to solidify the evidence on which physicians rely to treat critically ill patients. With such knowledge, more physicians will be able to adhere to the dictum “first, do no harm,” and we will have a surplus of blood for transfusion rather than a shortage.

## Conclusion

Clinicians of this hospital were conservative in keeping with recent transfusion guidelines. 21.4% of PRC transfusions were inappropriate and were required for patients with Hb above 9g%. 14.46% of FFP and 19% of platelet transfusions were inappropriately indicated. Blood or blood component request forms were still incomplete with the baseline investigations not mentioned in the request forms. However, a “look back” policy to educate the clinicians has reduced the rate of incomplete request forms. Increasing patient awareness demands that blood transfusion needs to be clinically justified.
